# Risk factors and potentials for fostering mental health and wellbeing in urban space

**DOI:** 10.1093/eurpub/ckac129.395

**Published:** 2022-10-25

**Authors:** H Müller, J Rehn-Groenendijk, A Wasmer

**Affiliations:** 1 Social and Cultural Sciences and Social Work, Darmstadt University of Applied Sciences, Darmstadt, Germany; 2 Design Research, Darmstadt University of Applied Sciences, Darmstadt, Germany; 3 Civil Engineering, Darmstadt University of Applied Sciences, Darmstadt, Germany

## Abstract

People shape their physical environments - and vice versa. As such, cities provide both resources (e.g., job opportunities, cultural diversity) as well as stressors (e.g., crowding, noise pollution) to their residents and visitors. In this context, numerous studies illustrate a considerable influence of the built environment (townscape, architecture) on health and well-being of interacting people. This impact ranges from physical aspects (e.g., traffic safety, particulate matter) to psychological processes (e.g., stress, loneliness) and behavioral aspects (e.g., physical activity, social behavior). At the same time, phenomena such as homelessness, crime, or mental disorders (e.g., substance addictions, schizophrenia) occur more frequently in cities compared to rural areas, illustrating causal as well as selective processes in the relation of urban environment and mental health. Increasing overall incidences in mental disorders (especially anxiety disorders and depression), the short-term shortage of psychotherapeutic care as well as the long-term economic burden on the health care system ask for a twofold strategy in public health: a) an extension of preventive measures with low threshold, i.e., accessible by large shares of the population, b) an extension of mental health literacy, which will empower the population to be attentive to mental health issues in themselves and others and which in turn can help to reduce stigmatization. While urban green and blue spaces have been researched in terms of restorative environments - allowing to regenerate resources consumed during the day - the built environment is still a resource for this strategy that has received insufficient attention to date. Utilizing the urban built environment not only as restorative but also informative and engaging environments thus affords an opportunity to address and potentially foster mental health and mental health literacy in citizens across socioeconomic backgrounds.

